# A cross-sectional study of characteristics of bicyclist upper and lower extremity injuries in bicycle-vehicle crashes in Ohio, United States, 2013–2017

**DOI:** 10.1186/s12889-021-10452-1

**Published:** 2021-03-02

**Authors:** Jodie Makara, Sijun Shen, Ann Nwosu, William Arnold, Gary Smith, Motao Zhu

**Affiliations:** 1 College of Medicine, The Ohio State University, Columbus, OH USA; 2 The Center for Injury Research and Policy, Abigail Wexner Research Institute at Nationwide Children’s Hospital, Columbus, OH USA; 3 Department of Pediatrics, College of Medicine, The Ohio State University, Columbus, OH USA; 4 Division of Neuromuscular Disorders Department of Neurology, Wexner Medical Center, The Ohio State University, Columbus, Ohio USA; 5 Department of Physical Medicine and Rehabilitation, Wexner Medical Center, The Ohio State University, Columbus, OH USA; 6 Division of Epidemiology, College of Public Health, The Ohio State University, Columbus, OH USA

**Keywords:** Accidents, Bicycling, Fractures, Hospital records

## Abstract

**Background:**

Extremity injury is one of the most common injury types for bicyclists. Extremity injury can lead to long-term disability and contribute to adverse health-related quality of life and prolonged absence from work.

**Objectives:**

The objectives of our study were to identify crash factors associated with bicyclist upper and lower extremity injury and characterize type of extremity injury by bicyclist age category.

**Methods:**

We linked the 2013–2017 Ohio police accident report and hospital databases. The logistic regression model was used to model the odds of sustaining upper or lower extremity injury among bicyclists involved in bicycle-vehicle crashes. Bicyclist upper and lower extremity injury were further described by the detailed injured body regions (e.g., forearm and elbow or lower leg) and the nature of injury (e.g., superficial or fracture).

**Results:**

Bicyclists 65 years or older had higher odds (odds ratio [OR] = 1.46, 95% confidence interval [CI]: 1.03–2.08) of sustaining upper extremity injury, bicyclists aged 3–14 years (OR = 1.34, 95% CI: 1.09–1.66) and 15–24 years (OR = 1.24, 95% CI: 1.03–1.49) had higher odds of sustaining lower extremity injury, compared to bicyclists 25–44 years old. In addition, colder weather, bicyclist sex, and intersection-related crashes were associated with bicyclists’ odds of sustaining upper or lower extremity injury. Compared to individuals under 65 years old, bicyclists 65 years or older had a higher percentage of injury to the wrist, hand and finger, or knee. Bicyclists aged 65 years or older also had a higher percentage of fractures.

**Conclusions:**

Our study has identified important factors that were associated with bicyclists’ odds of sustaining an extremity injury. Based on these findings, targeted educational efforts and interventions can be implemented to prevent bicyclists from these injuries.

## Introduction

Bicycle riding is a common mode of transportation and a popular recreational activity. The number of bicycle commuters increased by 43% from 2000 to 2017 in the United States (U.S.) [1]. Despite the increased safety efforts across the globe, the U.S. has made the least progress in reducing severe injury and fatality rates per kilometer among bicyclists compared to other countries [2]. It is well recognized that involvement of a motor vehicle in bicycle crashes increases severity of bicyclists’ injury, hospital healthcare cost, and length of hospital stay [3, 4]. In 2017, 96% of bicyclist crash-related deaths in the U.S. occurred in a bicycle-vehicle crash [5]. From 2002 to 2009 in the U.S., bicyclist injuries in bicycle-vehicle crashes averaged $425 million in hospital expenses per year [4]. They cost $23,424 more per visit and were over two times more likely to receive a nonroutine hospital discharge (e.g., death, transferring to nursing facility, and short-term hospital) compared to non-bicycle-vehicle injuries [4].

Many previous studies have investigated factors that contribute to bicyclists’ injury severity. Using police accident report (PAR) data, those studies suggested that higher speed limit, adverse weather conditions, darkness, head-on collisions, rush hours, and intersection-related crashes were associated with increased likelihoods for bicyclists to sustain severe injuries [6–8]. Male bicyclists, bicyclists at older age, male drivers, and drivers at younger age were also identified as significant contributing factors to the severity of bicyclists’ injuries [9]. However, the injury severity coded in PAR data was measured on the KABCO scale (i.e., no injury, possible injury, non-incapacitating injury, incapacitating injury, and fatal injury). Such a police-coded KABCO injury designation system does not require police to provide a strict and a comprehensive medical determination of the injury type and therefore, it is likely that internal or not immediately visible injuries may be overlooked by the police at the spot. Studies using PAR data to evaluate bicyclists’ injury severity often fail to account for specific injury details, such as the injured body region and the corresponding injury severity level coded by professional healthcare practitioners [10, 11].

Other studies have described bicyclist injuries in more detail using hospital records [12, 13]. For example, one study used data from eight hospital emergency departments in three states (California, New York, and North Carolina) and identified that bicyclists involved in bicycle-vehicle crashes were twice as likely to have a lower limb injury as riders involved in bicycle-only crashes [13]. However, studies using hospital record data have not evaluated bicyclist injuries with respect to other important crash factors, including roadway geometry, weather conditions, and type of collision. Thus, it is difficult for researchers to determine the important crash factors and thereby, suggest effective interventions to reduce the crash severity for bicyclists.

Different from previous studies, we used a database that probabilistically linked the Ohio statewide crash report with hospital records. This linked database provides us an opportunity to evaluate the relationship between crash factors and bicyclists’ injury details. To our knowledge, however, our study is among the first to utilize police-accident reported and hospital linked data to characterize crash factors related to specific body injury regions in bicyclists. The most common body regions injured in hospitalized bicyclists are the upper and lower extremities [14–16]. From a sample of 706 injured bicyclists at a Level One Trauma center in New York, 69 patients with lower extremity injuries required surgery, accounting for 48% of procedures, and patients with upper extremity accounted for 16% of procedures [16]. Although extremity injuries may not always be a life-threatening injury compared to other injuries (e.g., head injuries), they can lead to long-term disability and increased morbidity [14, 15]. One previous study identified that shoulder/upper arm injuries and lower limb injuries (mainly hip, thigh, and lower leg) contributed to adverse health-related quality of life and prolonged absence from work and school [17]. The objective of this study was to determine the associations of crash factors to upper and lower extremity injuries of the bicyclists involved in bicycle-vehicle crashes using the 2013–2017 Ohio crash report and hospital record linked data. Additionally, our study further specified bicyclists’ upper and lower extremity injuries by detailed region (e.g., shoulder and upper arm or forearm and elbow) and injury type (e.g., fracture or open wound).

## Methods

### Dataset

The 2013–2017 Ohio crash reports and Ohio hospital dataset were probabilistically linked for use. For developing the linked database, we obtained the police recorded crash data from the Ohio Department of Public Safety and the hospitalization data from the Ohio Hospital Association. Missing values in crash data were imputed using multivariate imputation by chained equations as implemented in IVEware [18]. Patient data included up to 15 diagnoses coded in the International Classification of Diseases-Ninth Revision (ICD-9 CM) or -Tenth Revision (ICD-10 CM) according to the year of hospitalization. Crash records were linked to patient data using probabilistic record linkage, which is a method that defines a series of linkage rules against which comparisons are drawn across multiple fields, including age, sex, date of birth, and date of the crash. The linkage process is described in further detail elsewhere [19]. This study was approved by the Research Institute of Nationwide Children’s Hospital’s Institutional Review Board, and the participant informed consent form was waived for our study.

In total, 6714 bicyclists in bicycle-vehicle crashes were identified. The vehicle types were limited to passenger car, minivan, sport utility vehicle (SUV), light trucks, and large trucks. The study population was further reduced to include bicyclists involved in a crash with only one moving motor vehicle. Cases of bicycle-vehicle crashes that involved a driver that was younger than 16 years of age, or a bicyclist that was younger than 3 years of age were excluded. As a result, 6451 bicyclists met our criteria. Among those bicyclists, 3842 (59.6%) were successfully linked with hospital records using our probabilistic model. The remaining unlinked bicyclists were those that either were not injured severely enough to be transported to the hospital following a crash or had incomplete matched information that hindered the ability to link them with crash reports. However, our linkage package was able to detect 89% true links that exist statewide [20], and therefore, most of the unlinked bicyclists were those who were not severely injured enough to have medical records. In total, among the 3842 bicyclists, 2600 bicyclists’ highest level of care (HLC) was in emergency department, 794 bicyclists’ HLC was in outpatient, and 448 bicyclists’ HLC was in inpatient. Regardless of their HLC, every linked bicyclist was provided up to 15 diagnoses coded in ICD-9 or ICD-10.

Bicyclists’ age was categorized as 3–14, 15–24, 25–44, 45–64, and 65+ years, and drivers’ age was classified into 16–24, 25–44, 45–64, and 65+ years. Vehicle body type was coded into three groups: 1) passenger car, 2) light truck, passenger van, SUV, and 3) large truck. Rain, sleet, snow, and fog were coded as adverse weather conditions as opposed to non-adverse weather conditions, which included clear and cloudy weather. Crashes that occurred between 6:00 am-5:59 pm were classified into daytime crashes, and otherwise they were coded as nighttime crashes [21]. The crash period was also grouped into weekday (between 6:00 am Monday and 5:59 pm Friday) and weekend (between 6:00 pm Friday and 5:59 am Monday) [21]. In addition, crashes that occurred from 7:00–9:59 am and 3:00–6:59 pm were classified as rush-hour crashes, and other crashes were coded as non-rush-hour crashes. The quarter-of-crash was coded as: quarter 1: January to March, quarter 2: April to June, quarter 3: July to September, and quarter 4: October to December.

Bicyclist injuries were either coded in ICD-9 CM or ICD-10 CM. For injuries coded in ICD-9 CM, the Barell injury diagnosis matrix was applied to identify the injured body region (e.g., upper or lower extremity) and the nature of injury (e.g., fracture, sprain/strain, superficial, or open wound) [22]. For injures coded in ICD-10 CM, the injured body region and nature of injury diagnoses were identified using the Injury Diagnosis Matrix provided by the National Center for Health Statistics [23]. Extremity injuries were further specified according to region of injury. Using the injury matrix framework, upper extremity referred to the shoulder and upper arm, forearm and elbow, wrist, hand, and fingers, and all other regions of the upper limbs (e.g., ill-defined fractures of upper limb). Lower extremity injuries included hip, upper leg and thigh, knee, lower leg and ankle, foot and toes, and all other regions of the lower limbs (e.g., other multiple and ill-defined fractures of lower limb). Extremity injuries were not mutually exclusive, and a single cyclist could sustain multiple injuries.

### Statistical analysis

Two binary logistic regression models were used to estimate the odds of injury to bicyclists’ upper or lower extremities. Logistic regression is a commonly used model in transportation safety that can describe the relationships between the crash factors and crash outcomes (e.g., injury odds/injury severity) [24]. A binary logistic regression is appropriate to apply when the dependent variable is dichotomous [25]. As the dependent variables in our models can take on only two values (1 = bicyclists sustaining upper extremity injury and 0 = bicyclists without upper extremity injury, or 1 = bicyclists sustaining lower extremity injury and 0 = bicyclists without lower extremity injury), the binary logistic regression is proper to use for our study.

The observations were the bicyclists who were successfully linked with a hospital record (in total, 3842 out of 6451 were linked). The dependent variables were the binary variables for the presence or absence of upper or lower extremity injuries. The independent variables were bicyclist age category (3–14, 15–24, 25–44, 45–64, or 65+ years), bicyclist sex (male or female), driver age category (16–24, 25–44, 45–64, or 65+ years), driver sex (male or female), vehicle body type (passenger car, van and SUV, or large trucks), vehicle model year (≤ 1999, 2000–2006, 2007–2011, or ≥ 2012), number of bicyclists involved in a crash (single-bicyclist crash or multiple-bicyclist crash), intersection-related crash (intersection-related crash or not intersection-related crash), weather condition (adverse weather or non-adverse weather conditions), time-of-day (daytime or nighttime), day-of-week (weekend or weekday), time-of-travel (rush hour or non-rush hour), quarter-of-crash (quarter 1, 2, 3, or 4), and year-of-crash (year 2013, 2014, 2015, 2016, or 2017). The backwards elimination procedure was utilized to identify the subset of variables providing the lowest Akaike’s Information Criterion (AIC) values (i.e., the best model fit).

The detailed injured regions of extremities were further subdivided and characterized by bicyclist age category. Upper extremity injuries were subdivided into three regions: 1) shoulder and upper arm, 2) forearm and elbow, and 3) wrist, hand and fingers. Lower extremity injuries were classified into five regions: 1) hip, 2) upper leg and thigh, 3) knee, 4) lower leg and ankle, and 5) foot and toes. The nature of injury to the upper and lower extremities was also described as 1) fracture, 2) open wound, or 3) superficial injury. The percentage of each injured body region or type of injury by each age category was calculated as the proportion of the number of bicyclists sustaining the corresponding injury out of the total number of bicyclists identified in crash reports in that age category. Statistical analyses were performed with SAS 9.4 software using PROC GENMOD and PROC MIANALYZE to combine results across imputations [26].

## Results

In total, 6451 bicyclists were involved in a single-vehicle crash with a passenger vehicle or truck in Ohio from 2013 to 2017. The sample characteristics are shown in Table 1. The average age for the bicyclists was 29.3 years (standard deviation [SD] = 8.2). Among all bicyclists, 96% were younger than 65 years. More than 80% of bicyclists were male. There were 3% of bicyclists who experienced bicycle-vehicle crashes involving multiple bicycles. Almost 54% of bicyclists were involved in a bicycle-vehicle crash with a male driver. According to the time-of-travel, about 40% of bicyclists were involved in a crash during rush hour. Most of our observations occurred in months that are generally warmer (quarters 2 and 3 accounted for 31 and 42% versus 8 and 19% in quarters 1 and 4, respectively). Table 2 presents the distribution of the injury types for the bicyclists that were successfully linked to their hospital records. Among those bicyclists, almost 38 and 51% of bicyclists sustained upper or lower extremity injury, respectively, suggesting extremity injury is a major injury type for bicyclists involved in bicycle-vehicle crashes.

**Table 1:** Characteristics for bicyclists involved in bicycle-vehicle Crashes in Ohio, United States, 2013–2017

Characteristic	N	Percent (%)
Bicyclist age category, year		
3–14 years	1598	24.8
15–24 years	1843	28.6
25–44 years	1420	22.0
45–64 years	1331	20.6
65+ years	259	4.0
Bicyclist sex		
Male	5229	81.1
Female	1222	18.9
Driver age category, year		
16–24 years	1051	16.3
25–44 years	2267	35.1
45–64 years	2109	32.7
65+ years	1024	15.9
Driver sex		
Male	3475	53.9
Female	2976	46.1
Vehicle body style		
Passenger car	3680	57.0
Light truck, passenger van, SUV	2708	42.0
Large truck	63	10.0
Vehicle model year		
≤ 1999	816	12.6
2000–2006	2442	37.9
2007–2011	1746	27.1
≥ 2012	1447	22.4
Number of bicyclists		
Single-bicyclist crash	6259	97.0
Multiple-bicyclist crash	192	3.0
Intersection related		
Not intersection related	2560	39.7
Intersection related	3891	60.3
Weather condition		
Non-adverse weather condition	5985	92.8
Adverse weather condition	466	7.2
Time-of-day		
Daytime (6:00 am-5:59 pm)	5092	78.9
Nighttime (otherwise)	1359	21.1
Time-of-travel		
Rush hour (7 am-9:59 am and 3 pm-6:59 pm Mon-Fri)	2577	39.9
Non-rush hour (otherwise)	3874	60.1
Day-of-week		
Weekend (6 pm Fri-5:59 am Mon)	1679	26.0
Weekday (otherwise)	4772	74.0
Quarter		
Quarter 1(January–March)	507	7.9
Quarter 2 (April–June)	2006	31.1
Quarter 3 (July–September)	2735	42.4
Quarter 4 (October–December)	1203	18.6
Year-of-crash		
2013	1317	20.4
2014	1315	20.4
2015	1265	19.6
2016	1280	19.8
2017	1274	19.7
Total No. bicyclists	6451	100.0

Data source: Ohio Department of Public Safety

**Table 2:** Body regions of injury for linked bicyclists

Body injury regions	N	Percent (%)
Traumatic brain injury	477	12.4
Other head, face, neck	1031	26.8
Spinal cord	20	0.5
Vertebral column	344	9.0
Torso	780	20.3
Upper extremity	1453	37.8
Lower extremity	1963	51.1
Unclassifiable by site	473	12.3
Total No. linked bicyclists	3842	100.0

### Odds of upper and lower extremity injury

Among the 3842 bicyclists that had linked hospital records using our probabilistic model, 1453 bicyclists sustained upper extremity injuries. The results of the logistic regression model for odds of upper extremity injuries among bicyclists involved in a bicycle-vehicle crash are presented in Table 3. Controlling for other important factors, the odds of an upper extremity injury among older adult cyclists were 1.46 times higher (95% confidence interval [CI]: 1.03–2.08) than that of bicyclists aged 25–44 years. The odds of an upper extremity injury in quarters 1 and 4 were significantly lower than in quarter 3 (odds ratio [OR] = 0.65, 95% CI: 0.49–0.87, and OR = 0.66, 95% CI: 54–0.81, respectively). Variation in the odds of an upper extremity injury were observed across time, with a significant increase in the odds of an upper extremity injury in 2016 (OR = 1.26, 95% CI: 1.02–1.56) and 2017 (OR = 1.36, 95% CI: 1.09–1.70) compared to 2013.

**Table 3:** Multivariable logistic regression model for odds of upper extremity injury among bicyclists in Ohio, 2013–2017

Variable	N (%)	Crude estimate (standard error)	Crude odds ratio (95%CI) ^**a**^	Adjusted estimate (standard error)	Adjusted odds ratio (95% CI) ^**b**^
Bicyclist age category, years					
3–14	343 (36.4)	0.04 (0.10)	1.04 (0.85–1.27)	−0.04 (0.11)	0.96 (0.78–1.18)
15–24	418 (38.1)	0.11 (0.10)	1.12 (0.93–1.35)	0.08 (0.01)	1.08 (0.89–1.31)
25–44	296 (35.5)	-- ^c^	-- ^c^	-- ^c^	--^c^
45–64	323 (39.8)	0.18 (0.11)	1.20 (0.98–1.48)	0.17 (0.11)	1.19 (0.97–1.47)
65+	73 (46.1)	0.44 (0.18) *	**1.56 (1.10–2.21)**	0.38 (0.18) *	**1.46 (1.03–2.08)**
Weather condition					
Non-adverse weather condition	1364 (38.3)	-- ^c^	--^c^	-- ^c^	--^c^
Adverse weather condition	89 (31.6)	−0.30 (0.14) *	**0.74 (0.57–0.97)**	− 0.21 (0.14)	0.81 (0.62–1.07)
Time-of-day					
Daytime	1154 (38.8)	-- ^c^	--^c^	-- ^c^	--^c^
Nighttime	299 (34.6)	−0.19 (0.08) *	**0.83 (0.71–0.98)**	−0.07 (0.09)	0.93 (0.78–1.11)
Quarter					
Quarter 1(January–March)	84 (30.7)	−0.42 (0.14) **	**0.66 (0.50–0.87)**	−0.43 (0.15) **	**0.65 (0.49–0.87)**
Quarter 2 (April–June)	482 (40.4)	0.00 (0.08)	1.00 (0.86–1.17)	0.02 (0.08)	1.02 (0.87–1.19)
Quarter 3 (July–September)	667 (40.3)	-- ^c^	--^c^	-- ^c^	--^c^
Quarter 4 (October–December)	220 (30.5)	−0.43 (0.10) **	**0.65 (0.53–0.79)**	−0.42 (0.10) **	**0.66 (0.54–0.81)**
Year-of-crash					
2013	267 (34.0)	-- ^c^	--^c^	-- ^c^	--c
2014	304 (37.9)	0.17 (0.11)	1.19 (0.96–1.47)	0.14 (0.11)	1.15 (0.92–1.43)
2015	263 (36.8)	0.12 (0.12)	1.13 (0.89–1.42)	0.12 (0.12)	1.13 (0.89–1.43)
2016	304 (39.1)	0.22 (0.11) *	**1.25 (1.01–1.54)**	0.23 (0.11) *	**1.26 (1.02–1.56)**
2017	315 (41.2)	0.31 (0.11) **	**1.36 (1.09–1.69)**	0.31 (0.11) **	**1.36 (1.09–1.70)**

Note: ^a^ Odds ratio calculated from the logistic regression model with a single explanatory variable

^b^ Odds ratio calculated from the multivariable logistic regression model including covariates for bicyclist age category, adverse weather conditions, time-of-day, quarter, and year-of-crash

^c^ refers to reference group

Data sources: Ohio Hospital Association and Ohio Department of Public Safety

Bolded values indicate the 95% confidence interval excludes 1

* refers the *p* value < 0.05

** refers the *p* value < 0.01

The number of bicyclists that sustained lower extremity injuries was 1963. The odds ratios for lower extremity injuries among bicyclists who were treated in a hospital following a crash are presented in Table 4. Adjusting for important driver and crash characteristics, among bicyclists aged 3–14 years and 15–24 years, the odds of a lower extremity injury were 1.34 and 1.24 times that of bicyclists aged 25–44 years (95% CI: 1.09–1.66, and 95% CI: 1.03–1.49, respectively). Additionally, female bicyclists had a higher odds of a lower extremity injury (OR = 1.19, 95% CI: 1.01–1.41) relative to males. The odds of sustaining a lower extremity injury at an intersection for a bicyclist was 1.16 times (95% CI: 1.01–1.33) that of non-intersection crashes.

**Table 4:** Multivariable logistic regression model for odds of lower extremity injury among bicyclists in Ohio, 2013–2017

Variable	N (%)	Crude estimate (standard error)	Crude odds ratio (95%CI) ^**a**^	Adjusted estimate (standard error)	Adjusted odds ratio (95% CI) ^**b**^
Bicyclist age category, years					
3–14	514 (54.7)	0.27 (0.10) **	**1.31 (1.07–1.60)**	0.29 (0.11) **	**1.34 (1.09–1.66)**
15–24	583 (53.1)	0.21 (0.09) *	**1.23 (1.02–1.47)**	0.22 (0.09)	**1.24 (1.03–1.49)**
25–44	400 (48.0)	-- ^c^	-- ^c^	-- ^c^	-- ^c^
45–64	392 (48.4)	0.02 (0.10)	1.02 (0.83–1.24)	0.05 (0.10)	1.05 (0.86–1.28)
65+	74 (46.5)	−0.06 (0.17)	0.94 (0.67–1.33)	− 0.01 (0.18)	0.99 (0.70–1.40)
Bicyclist sex					
Male	1552 (50.2)	-- ^c^	-- ^c^	-- ^c^	-- ^c^
Female	411 (54.7)	0.17 (0.09) *	**1.19 (1.01–1.41)**	0.69 (0.09) **	**1.19 (1.01–1.41)**
Driver sex					
Male	1087 (52.1)	-- ^c^	-- ^c^	-- ^c^	–
Female	876 (49.9)	−0.08 (0.07)	0.92 (0.80–1.05)	− 0.09 (0.07)	0.91 (0.79–1.05)
Intersection related					
Not intersection related	751 (48.9)	-- ^c^	-- ^c^	-- ^c^	-- ^c^
Intersection related	1212 (52.5)	0.14 (0.07) *	**1.15 (1.00–1.33)**	0.15 (0.07) *	**1.16 (1.01–1.33)**
Time-of-day					
Daytime	1514 (50.9)	-- ^c^	-- ^c^	-- ^c^	-- ^c^
Nighttime	449 (51.8)	0.04 (0.09)	1.04 (0.88–1.23)	0.07 (0.09)	1.07 (0.91–1.27)

Note: ^a^ Odds ratio calculated from the logistic regression model with a single explanatory variable

^b^ Odds ratio calculated from the multivariable logistic regression model including covariates for cyclist age group, cyclist sex, driver sex, intersection related crash, and time-of-day

^c^ refers to reference group

Data sources: Ohio Hospital Association and Ohio Department of Public Safety

Bolded values indicate the 95% confidence interval excludes 1

* refers the *p* value < 0.05

** refers the *p* value < 0.01

### Extremity injury patterns by age category

**Fig. 1:**
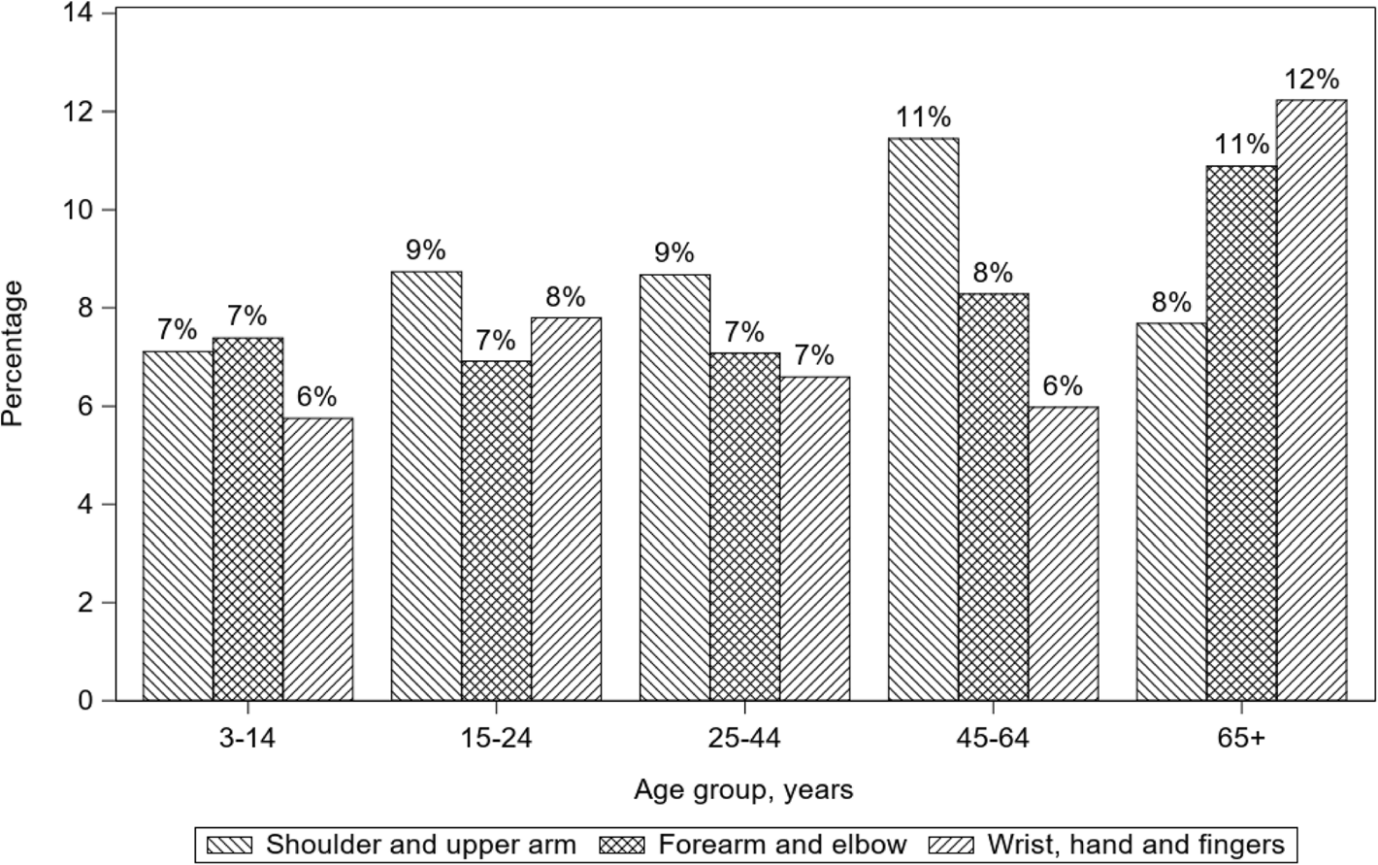
Type of upper extremity injury stratified by bicyclist age category

Specified upper extremity injuries to the shoulder and upper arm, forearm and elbow, and wrist, hand and fingers were stratified by bicyclist age category (Fig. 1). Bicyclists 65 years or older had higher percentages of sustaining injuries to the forearm and elbow and wrist, hand, and fingers in a crash than other age categories. For bicyclists younger than 45 years, the percentages of shoulder and upper arm, forearm and elbow, and wrist, hand and fingers injuries ranged between 6 and 9% and were evenly distributed. However, for bicyclists aged 45–64 years, the percentage of shoulder and upper arm injury was 11%, which was larger than the percentages of other specific upper extremity regions. In contrast, the percentage of shoulder and upper arm injuries for bicyclists aged 65 years or older was 8%, which was smaller than the percentages of forearm and elbow (11%) and wrist, hand, and fingers (12%) injuries.

**Fig. 2:**
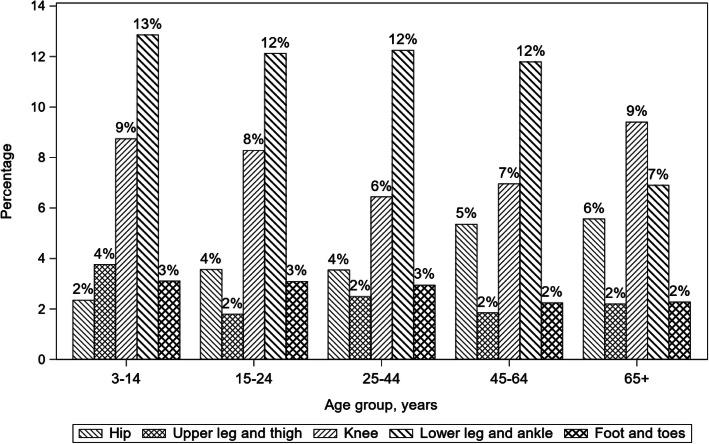
Type of lower extremity injury stratified by bicyclist age category

Lower extremity injuries to the foot and toes, hip, upper leg and thigh, knee, and lower leg and ankle injuries were also stratified by bicyclist age category (Fig. 2). The lower leg and ankle were the dominant lower extremity injured regions among bicyclists younger than 65 years old, followed by the knee. At least 12% of bicyclists younger than 65 years old sustained lower leg and ankle injuries. In contrast, among bicyclists aged 65 years and older, the dominant region of lower extremity injury was the knee (9%), followed by the lower leg and ankle (7%).

The nature of extremity injury among bicyclists involved in motor vehicle crashes was also examined (Table 5). Superficial injuries to the extremities were sustained by more than 10% of bicyclists and this injury was the most common injury type seen in all age categories. Although fracture of the upper or lower limbs was commonly reported for all age categories, fractures accounted for the greatest proportion of injuries among bicyclists aged 65 years or older. Fracture to the upper or lower extremity was reported among 9.7 and 8.1%, respectively, of bicyclists aged 65 years or older involved in bicycle-vehicle crashes. The proportion of open wound injuries also increased with increasing bicyclist age group. The percentage of open wound injury to the upper extremities increased from 2.1% among bicyclists aged 45–64 years to 6.6% among bicyclists aged 65 years or older.

**Table 5:** Nature of upper and lower extremity injury stratified by bicyclist age group

	Age category, years				
	3–14	15–24	25–44	45–64	65+
Upper extremity					
Fracture	62 (3.9)	78 (4.2)	71 (5.0)	98 (7.4)	25 (9.7)
Open wound	18 (1.1)	40 (2.2)	29 (2.0)	28 (2.1)	17 (6.6)
Superficial	233 (14.6)	258 (14.0)	164 (11.5)	177 (13.3)	36 (13.9)
Lower extremity					
Fracture	86 (5.4)	68 (3.7)	80 (5.6)	105 (7.9)	21 (8.1)
Open wound	47 (2.9)	55 (3.0)	34 (2.4)	31 (2.3)	9 (3.5)
Superficial	343 (21.5)	376 (20.4)	239 (16.8)	246 (18.5)	48 (18.5)
Total number of bicyclists	1598	1843	1420	1331	259

Note: An individual cyclist may have multiple injuries

Data sources: Ohio Hospital Association and Ohio Department of Public Safety

## Discussion

The objective of our study was to determine the factors that contribute to risk of upper and lower extremity injury in bicyclists involved in bicycle-vehicle crashes. Approximately half of bicyclists who were transferred to a hospital following a bicycle-vehicle crash sustained upper and/or lower extremity injuries. Our results are consistent with the findings of previous studies that upper and lower extremity injuries were the most common type of injuries sustained by bicyclists [14–16].

Our results found that, compared to bicyclists aged 25–44 years, bicyclists 65 years or older had higher odds of sustaining upper extremity injuries, and bicyclists aged 3–14 and 15–24 had higher odds of sustaining lower extremity injuries. Older adults are more likely to have weak bones and thus low impact fractures as compared with younger adults and children [27]. With a farther distance to fall, the upper extremity is more susceptible to injury. Thus, upper extremity protection (e.g., elbow pads and wrist guard) is particularly important for older bicyclists. In addition, a previous study identified that younger bicyclists were less skilled than the middle-aged counterparts in coordinating self and object movement [28], which could possibly explain why bicyclists aged 3–14 and 15–24 years were more likely to sustain lower extremity injury than bicyclists aged 25–44 years. Specific education and training programs for bicyclists aged 3–24 years could be considered to help prevent lower extremity injuries in this age group.

Seasonality also played a role in determining the odds of bicyclists sustaining upper extremity injury. Bicyclists in quarters 1 (January–March) and 4 (October–December) were less likely to have an upper extremity injury compared to quarter 3 (July–September). One possible reason is that in Ohio, during quarters 1 and 4, the temperature is much lower than quarter 3. Bicyclists may wear heavier clothing in colder weather. The heavier clothing could serve as a cushion and reduce impact between bicyclists and vehicles or with the ground, resulting in smaller odds of upper extremity injuries for bicyclists. Bicyclists also might ride at lower speeds during colder weather, reducing impact energy and odds of upper extremity injury.

Bicyclists involved in intersection-related crashes had higher odds of sustaining lower extremity injuries. Kinematics during the impact between bicyclists and vehicles at intersections may help explain bicyclists’ higher odds of sustaining lower extremity injuries [29]. During an intersection-related crash, bicyclists are more likely to be crossing paths with vehicles rather than riding alongside them. Therefore, their legs may be at higher risk of being struck from the side by the front of the vehicle. Previous studies have advocated that city planners and traffic safety professionals should identify risky intersections with respect to bicycle safety and improve the roadway design (e.g., optimize the signal timing at intersections) to prevent bicyclist injuries proactively [30].

Bicyclists aged 65 years or older had increased likelihoods of forearm/elbow and wrist/hand/finger injuries. One previous study has identified that the older bicyclists were more likely to sustain those types of injuries when falling from the bicycle on an outstretched upper limb [31]. This further suggests the importance of wearing elbow pads and wrist guard for older bicyclists.

In addition to upper extremity injuries, bicyclists aged 65 years or older had the highest rates of knee injuries compared to all other age categories. The knee is susceptible to injury due to the shallow bony socket formed by the femur and tibia. Therefore, the joint mainly relies on ligaments and muscle tendons for stability. Older adults, being at greater risk for osteoarthritis, may have osteophytes, cell senescence and age-related changes to the joint, resulting in heightened pain and more severe damage when a collision involves that region [32].

Bicyclists aged 65 years or older were more likely to sustain a fracture of the upper or lower extremities. With older individuals being at higher risks for osteoporosis [27], they are more likely to have fractures in bicycle-vehicle crashes. Older bicyclists involved with bicycle-vehicle crashes were also more likely to sustain open wound injury. This may be related to diminished integrity of the skin when subjected to sheer forces in older adults [33].

### Study limitations

Some notable limitations were associated with our study. First, every bicyclist included in our logistic models was linked to a hospital record. Bicyclists who are transported to a hospital generally have more severe injuries and need medical treatment relative to those who are not transported to a hospital and thereby, our results may not be able to represent the full burden of all bicycle-vehicle crashes. In addition, due to the missing or incomplete data, not all potential linked records can be captured by our linkage package, further limiting the generalizability of our results. Second, bicyclists in our study were from Ohio, and our results may not be applied to other states that may have significantly different weather and road conditions. Third, in general, extremity injury is not as life-threatening as other types of injury, including head and internal injury. Thus, bicyclists with higher odds of extremity injury did not necessarily have higher odds of sustaining a more severe injury. Future research should explore the relationships between crash factors and extremity injury severity for bicyclists, particularly when extremity injury is the dominate injury. Fourth, although we believed that we used the best method to probabilistically link the crash records with hospital history, they were not deterministic linkage and some mismatches between records might exist. The indeterministic linkage may result in bias of our analysis.

## Conclusions

By characterizing factors associated with extremity injuries among bicyclists in bicycle-vehicle crashes, findings from our study provide information to support specific education efforts and interventions to reduce bicyclists’ extremity injuries and improve bicyclist safety. Specifically, our findings suggest that more attention should be directed towards prevention of upper extremity injuries, especially fractures, among older adult bicyclists in bicycle-vehicle crashes. Safety campaigns are needed to improve older bicyclists’ use of bicycle safety equipment (e.g., elbow pads) to help mitigate the risk of fracture. For bicyclists 24 years or younger, bicycle skill training programs are necessary to improve their cycling skills and experience and reduce their odds of sustaining lower extremity injury. Seasonality also plays an important role in determining the odds of upper extremity injury. Bicyclists’ heavier clothing in colder seasons may act as a cushion to mitigate the vehicle-bicycle impact and prevent injuries, further suggesting the safety benefits of bicycle safety equipment to bicyclists. Bicyclists should be educated about the dangers of intersections and could benefit from an improved intersection design.

## Data Availability

The data that support the findings of this study are available from Ohio Department of Public Safety and Ohio Hospital Association, but restrictions apply to the availability of these data, which were used under license for the current study, and so are not publicly available. Data are however available from the authors upon reasonable request and with permissions of Ohio Department of Public Safety and Ohio Hospital Association.

## Abbreviations

AIC: Akaike’s Information Criterion
CI: Confidence interval
ICD-9: International Classification of Diseases-Ninth Revision
ICD-10: International Classification of Diseases-Tenth Revision
OR: Odds ratio
PAR data: Police accident reported data
SUV: Sport utility vehicle
SD: Standard deviation
U.S.: United States
